# Supplementary material for: Physiological Response of Lettuce (Lactuca sativa L.) Grown on Technosols Designed for Soil Remediation
Source: Plants (Basel). 2024 Nov 16;13(22):3222. doi: 10.3390/plants13223222 (PMC11598719; doi:10.3390/plants13223222)
Supplement: Supplementary file 1 [file plants-13-03222-s001.zip › plants-3205552-supplementary.pdf]

plants

MDPI

Supplementary Materials

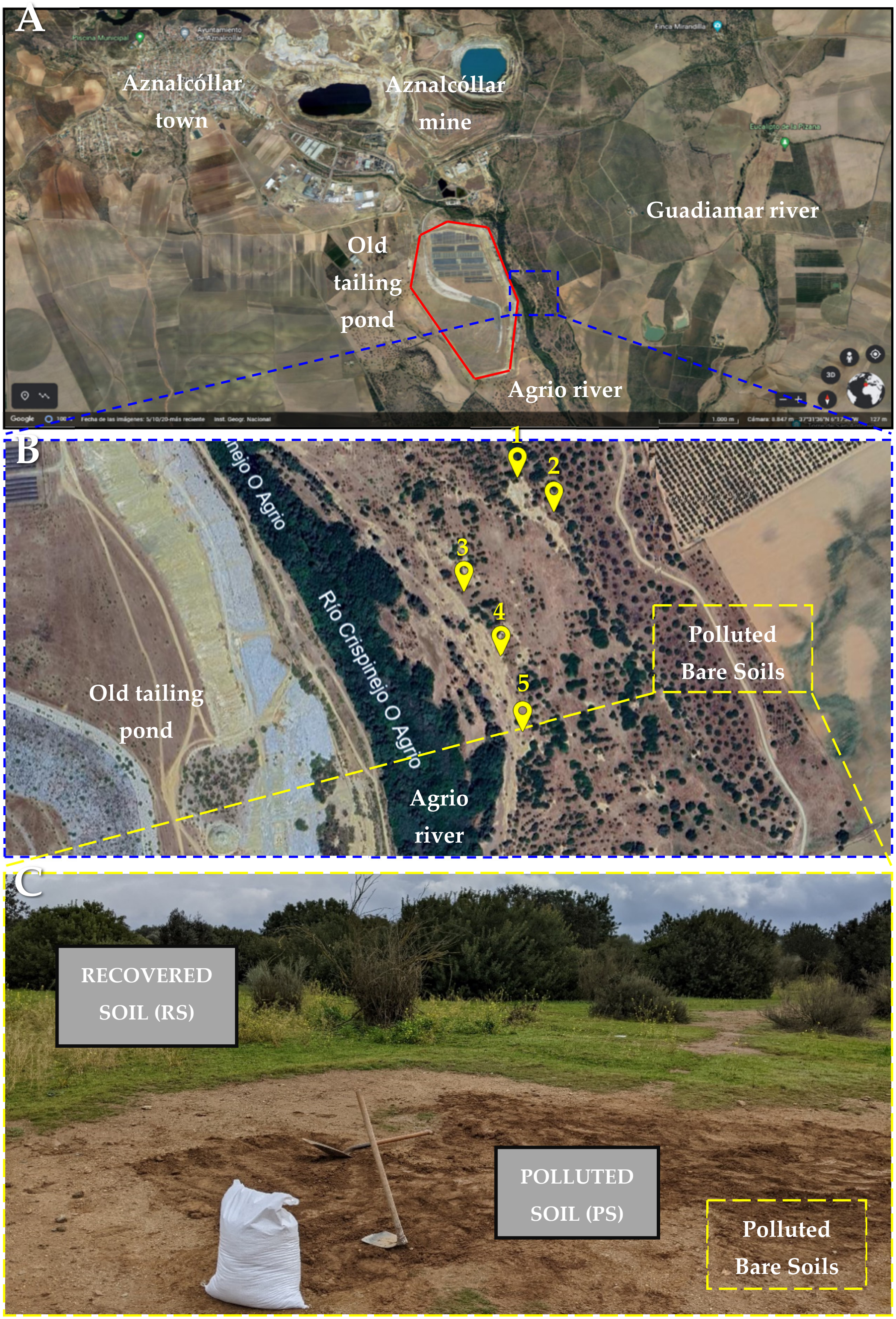

Figure S1. (A) Location of the Aznalcóllar mine and tailing pond that was breached. (B) Location of the five sampling locations (1: 37° 29′ 42′′ N, 6° 13′ 12′′ W; 2: 37° 29′ 40′′ N, 6° 13′ 10′′ W; 3: 37° 29′ 38′′ N, 6° 13′ 16′′ W; 4: 37° 29′ 35′′ N, 6° 13′ 13′′ W; 5: 37° 29′ 32′′ N, 6° 13′ 12′′ W). (C) Detail of the persistent polluted soils (PS), characterised by the absence of vegetation, and of the surrounding recovered soil (RS) in the vicinity of the old tailings pond of the Aznalcóllar mine.

Table S1. Main properties of the amendments used in the production of Technosols TO, TS, and TV (IO, MS, OL, WS, VC).

Waste	**pH** $_{1:2.5}$ **(H_2O)**	**EC** $_{1:5}$ (dS m^{-1})	**OC** (%)	**$CaCO_3$** (%)	**CEC** ($cmol_+$ kg^{-1})	**Ca^{2+}** ($cmol_+$ kg^{-1})	**Mg^{2+}** ($cmol_+$ kg^{-1})	**Na^+** ($cmol_+$ kg^{-1})	**K^+** ($cmol_+$ kg^{-1})	**N_T** (%)	**C_T** (%)	**Fe_T** (%)	**P_A** (mg kg^{-1})	**BR** (µg CO_2 day^{-1} kg^{-1})
**IO**	7.27 ± 0.08	0.04 ± 0.01	n.d.	13.68 ± 0.22	6.34 ± 0.38	4.44 ± 0.39	n.d.	1.11 ± 0.03	0.79 ± 0.01	n.d.	1.55 ± 0.01	95.99 ± 0.88	n.d.	45.91 ± 4.42
**MS**	8.27 ± 0.13	1.13 ± 0.08	0.16 ± 0.11	99.99 ± 0.01	9.34 ± 4.76	5.59 ± 4.60	1.44 ± 0.27	1.48 ± 0.13	0.84 ± 0.02	n.d.	11.81 ± 0.05	0.17 ± 0.01	n.d.	29.46 ± 0.20
**OL**	8.63 ± 0.03	3.67 ± 1.86	28.08 ± 1.76	20.96 ± 1.25	90.72 ± 3.57	6.54 ± 2.00	2.22 ± 0.13	8.81 ± 0.03	73.15 ± 1.75	1.63 ± 0.28	30.60 ± 6.90	0.62 ± <0.01	318.70 ± 98.25	70.70 ± 32.73
**WS**	7.16 ± 0.02	10.13 ± 0.69	21.95 ± 2.91	10.77 ± 0.92	55.11 ± 5.81	24.94 ± 1.07	9.02 ± 0.71	5.67 ± 0.31	6.35 ± 0.19	3.13 ± 0.09	23.37 ± 0.35	2.01 ± 0.09	401.53 ± 14.07	13.95 ± 1.73
**VC**	7.35 ± 0.08	0.39 ± 0.07	10.50 ± 4.08	24.87 ± 1.30	35.83 ± 2.16	23.25 ± 2.00	8.36 ± 0.24	1.20 ± 0.02	3.02 ± 0.04	0.64 ± 0.02	12.44 ± 0.18	0.98 ± 0.13	226.94 ± 37.65	82.96 ± 9.36

IO – Iron oxyhydroxide-rich sludge, MS – Marble cutting and polishing sludge, OL – Solid olive-mill by-product, WS – Composted sewage sludge, VC – Vermicompost from pruning and gardening, EC – Electric conductivity, OC – Organic carbon content, $CaCO_3$ - Calcium carbonate content, CEC - Cation exchange capacity, N_T/C_T/Fe_T - Total concentrations of N, C and Fe, P_A – Assimilable phosphorus, BR – Basal respiration rate, n.d. - not detected. Extracted from [43].

Table S2. Total concentrations of potentially harmful elements in mg kg^{-1} in the amendments used in the production of Technosols TO, TS, and TV (IO, MS, OL, WS, VC).

Waste	As	Cd	Co	Cr	Cu	Ni	Pb	Sb	V	Zn
**IO**	23.92 ± 1.98	0.03 ± 0.02	3.82 ± 0.52	6.18 ± 0.56	5.80 ± 0.28	6.96 ± 0.61	28.56 ± 2.94	20.85 ± 0.90	43.10 ± 2.88	25.86 ± 1.68
**MS**	0.77 ± 0.17	0.15 ± 0.02	1.21 ± 0.04	5.35 ± 0.18	4.01 ± 0.03	2.94 ± 0.09	2.44 ± 0.35	0.33 ± 0.09	5.39 ± 0.31	5.45 ± 0.80
**OL**	2.40 ± 0.73	0.06 ± 0.04	4.96 ± 1.02	16.29± 3.35	147.08 ± 26.73	12.27 ± 2.44	3.54 ± 0.84	0.27 ± 0.06	25.10 ± 4.51	62.86± 11.38
**WS**	5.26 ± 1.76	0.72 ± 0.18	3.88 ± 0.71	33.61 ± 9.35	211.83 ± 9.52	20.75 ± 2.07	39.79 ± 5.15	n.d.	26.79 ± 3.00	517.19 ± 67.54
**VC**	3.22± 0.42	0.19 ± 0.04	2.83 ± 0.36	24.05 ± 4.37	25.13 ± 2.60	13.20 ± 2.12	24.09 ± 2.71	n.d.	23.50 ± 3.44	156.08 ± 24.23

IO – Iron oxyhydroxide-rich sludge, MS – Marble cutting and polishing sludge, OL – Solid olive-mill by-product, WS – Composted sewage sludge, VC – Vermicompost from pruning and gardening, Extracted from [43].

Table S3. Results of the certified reference material of Loamy clay soil CRM 052–050 (RT-Corporation Limited, Salisbury, UK) analysed by ICP-MS in a PerkinElmer NexION 300D spectrometer (PerkinElmer, Inc., Waltham, MA, USA) after acid digestion in *aqua regia* in a Mars XP1500 Plus microwave (CEM Corporation, Matthews, CN, USA).

Element	Certified value	Prediction interval		Measured value
		Low	*High*	
**As**	14.60	10.90	18.30	13.00
**Cd**	35.60	31.60	39.60	33.49
**Cu**	44.20	37.60	50.80	38.20
**Fe**	12400	9870	14900	11190
**Pb**	82.60	71.80	93.40	75.30
**Zn**	89.00	70.90	107.00	77.85

Measured values are the mean of three replicates. All values are given in mg kg^{-1}.

Table S4. Results of the certified reference material Lichen BCR® - 482 (EC-JRC-IRMM, Geel, Belgium) analysed by ICP-MS in a PerkinElmer NexION 300D spectrometer (PerkinElmer, Inc., Waltham, MA, USA) after acid digestion in *aqua regia* in a Mars XP1500 Plus microwave (CEM Corporation, Matthews, CN, USA).

Element	Certified value	Measured value	Variance +/- (%)
**As**	0.85	0.73	- 14
**Cd**	0.56	0.43	- 23
**Cu**	7.03	6.57	- 7
**Pb**	40.9	33.75	- 17
**Zn**	100.6	76.75	- 24

Measured values are the mean of three replicates. All values are given in mg kg^{-1}.
